# Electrolysis and Its Application to the Treatment of Disease

**Published:** 1871-11

**Authors:** 


### Books Reviewed.

*Electrolysis and its Application to the Treatment of Disease.* By A. D. Rockwell, A.M., M.D.

In this pamphlet, which is a reprint from the New York Medical Journal, the author expresses a very favorable opinion as to the value of electrolysis in the treatment of many diseases. He says:

"When we come to inquire concerning the practical results yielded by electrolyzation in its application to tumors and other surgical diseases, the field opened before us is a most encouraging one. A great variety of abnormal growths, nævi and papillary enlargements, sebaceous, hydatid, and erectile tumors, goitres, and even cancers, are reported as having been successfully treated by electrolysis. Our own experience in this department of electro-therapeutics, not yet very extensive, has been on the whole measurably successful."

